# The potential clinical significance of immunomodulation of immune and inflammatory responses involving selected adipokines in patients with ovarian cancer – preliminary studies

**DOI:** 10.3389/fonc.2026.1757624

**Published:** 2026-04-14

**Authors:** Sebastian Stępień, Maria-Laura Morawiec, Marta Smycz-Kubańska, Andrzej Witek, Aleksandra Mielczarek-Palacz

**Affiliations:** 1 Department of Immunology and Serology, Faculty of Pharmaceutical Sciences in Sosnowiec, Medical University of Silesia, Katowice, Poland; 2 Department of Gynecology and Obstetrics, Faculty of Medical Sciences in Katowice, Medical University of Silesia, Katowice, Poland

**Keywords:** adipokines, chemerin, irisin, lipocalin-2, omentin-1, ovarian cancer

## Abstract

Ovarian cancer (OC) is a major clinical problem in gynaecological oncology, characterised by difficulties in early diagnosis, which contributes to the high mortality rate from OC. Therefore, the search for new biological markers continues, which could complement the diagnostic process in the future and potentially improve patient prognosis. Recently, the role of adipokines and accompanying inflammation in carcinogenesis mechanisms, including within the ovary, has become a subject of scientific interest. The aim of this study was to determine the concentrations of irisin, chemerin, lipocalin-2 and omentin-1 in the serum and peritoneal fluid of patients with ovarian cancer, as well as in the serum of patients with benign ovarian lesions. The analysis showed differences in the concentrations of these molecules between the study groups. Certain correlations were also observed between the levels of selected adipokines and the degree of histological differentiation of the tumour. The observed immune disorders associated with adipokine profiles suggest that they may be involved in the immunopathogenesis of ovarian cancer, acting as one of the factors modulating the inflammatory environment of the tumour. From a clinical point of view, the potential application of measuring the concentrations of these adipokines – for example, in combination with known markers such as CRP or CA125 – increases diagnostic effectiveness compared to single measurement of these parameters, but requires further verification. At this stage, they can only serve as a starting point for discussion on new diagnostic methods.

## Graphical Abstract

**Figure:**
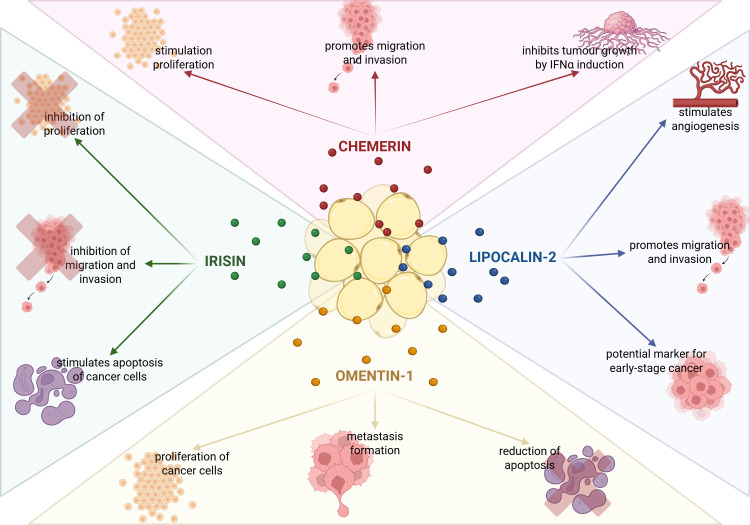
Schematic representation of the role of the analysed adipokines in ovarian cancer carcinogenesis. Created in https://BioRender.com

## 1 Introduction

Ovarian cancer (OC) is still a major problem in modern gynaecological oncology. The reason for this is that OC is a heterogeneous cancer characterised by diverse risk factors, precursor lesions, pathogenesis, patterns of spread, molecular profiles, clinical course, response to chemotherapy and treatment outcomes (1).

According to data from the Global Cancer Observatory (GLOBOCAN), the incidence and mortality rates for ovarian cancer remain high. The incidence rate in 2018, 2020 and 2022 was 1.6% (% of all sites), while the mortality rate in those years was 1.9%, 2.1% and 2.1% (% of all sites), respectively (2–4). Despite numerous studies, the exact mechanisms underlying the development and progression of ovarian cancer are not fully understood, as they result from complex interactions between cancer cells and the tumour microenvironment. These interactions include both direct and indirect interactions involving soluble mediators of the immune system – cytokines (5–7).

Adipokines constitute a significant group of these mediators. Adipokines are biologically active signalling molecules (cytokines) involved in regulating processes between the immune and endocrine systems (8). They play an important role in modulating the inflammatory process, as they can induce chronic inflammation and exhibit anti-inflammatory properties. An imbalance between these processes is responsible for immune system dysfunction, as well as local and systemic inflammation accompanying many cancers, including ovarian cancer (8, 9).

Cancer-associated adipocytes (CAA) play a special role in the microenvironment of ovarian cancer. They are characterised by abnormal energy metabolism (active lipolysis) and overexpression of cytokines and proteases, which promotes proliferation, invasion and resistance to treatment (10, 11). Characterising functional and phenotypic changes in CAA paves the way for identifying new molecular targets and developing strategies for modulating the tumour microenvironment. Since the immune system and adipokines present in the peritoneal cavity determine the dynamics of ovarian cancer development, understanding them is crucial for inhibiting tumour growth. Adipokines can play a dual role: they can serve as biomarkers for monitoring disease progression and provide a basis for designing precise therapeutic procedures targeting the mechanisms underlying carcinogenesis (10–12).

The role of adipokines in ovarian cancer remains unclear and is the subject of considerable controversy among researchers. On the one hand, molecules such as leptin, visfatin and resistin exhibit strong pro-tumour properties, stimulating tumour cell growth and invasiveness. On the other hand, adiponectin and omentin have suppressive functions, inhibiting angiogenesis and tumour migration processes (13, 14). Recent reports emphasise the importance of these substances in therapeutic processes. The potential use of inhibitors of selected signalling pathways in immunomodulation could significantly limit the expansion of cancer cells into the larger network. For this reason, adipocytokines are becoming a key starting point for targeted therapies, which are currently undergoing intensive analysis both *in vitro* and in clinical trials (15, 16). Despite promising results, the dynamic interactions between cancer cells and adipokines require further exploration. Current evidence suggests that their impact on disease progression is twofold: directly, through paracrine signalling, and indirectly, through modulation of the immune microenvironment (8, 9). It is therefore essential to precisely explain the mechanisms that lead to the modification of the phenotype of adipocytes and their biological activity, which will allow for a full understanding of the biology of this cancer (16).

Irisin, chemerin, lipocalin-2 and omentin-1 are examples of adipokines involved in the immune response in ovarian cancer. Moreover, on the one hand, they can enhance the anti-tumour response, but on the other hand, they can also promote tumour progression, which makes them extremely interesting proteins in the context of potential clinical applications, including immunodiagnostics and immunotherapy of tumours (17–20).

Research is ongoing to understand the role of irisin in many types of malignant tumours, but the results obtained are inconsistent and require further analysis (19, 21). However, studies conducted on *in vitro* models provide important clues about the mechanisms of action of this molecule. Irisin has been shown to inhibit the proliferation, migration and invasiveness of cells in lung cancer. As a result, this molecule can reverse the epithelial-mesenchymal transition (EMT) process, which directly limits the tumour’s ability to metastasis (22). Similar relationships have been observed in studies on pancreatic cancer. Irisin induces apoptosis in these cells and inhibits their proliferation in a dose-dependent manner. In addition, it limits the migratory and invasive abilities of the tumour, which allows it to be considered a potential target for new therapeutic strategies (23). Reports on osteosarcoma indicate that irisin is involved in inhibiting processes that promote an aggressive cell phenotype, including changes related to their ability to metastasise (24).

Available data indicate that chemerin also known as retinoic acid receptor responder 2 (RARRES2), depending on the type of cancer, can exhibit both anti-tumour and pro-tumour effects. It is suggested that its dual role in carcinogenesis stems from its dual role in inflammation, as a pro-inflammatory and anti-inflammatory mediator (17, 25–27). The pro-tumour role of chemerin has been demonstrated in squamous cell carcinoma of the oral cavity (28), gastric cancer (26), prostate cancer (29), while its anti-tumour role has been demonstrated in melanoma (30). In the case of ovarian cancer, the effect of chemerin on its development is ambiguous – on the one hand, chemerin has an anti-cancer effect, while on the other hand, it promotes tumour growth (15).

Recent studies show that lipocalin-2 (LCN-2) also known as neutrophil gelatinase associated lipocalin (NGAL) plays a role in the development, proliferation and invasion of tumours (31, 32). It has been demonstrated to play a role in the initiation, promotion and progression of colorectal cancer (33), breast cancer (34), prostate cancer (35) and endometrial cancer (36). In addition, there are also studies indicating the role of NGAL in ovarian cancer (18, 37–40).

**Figure 1:**
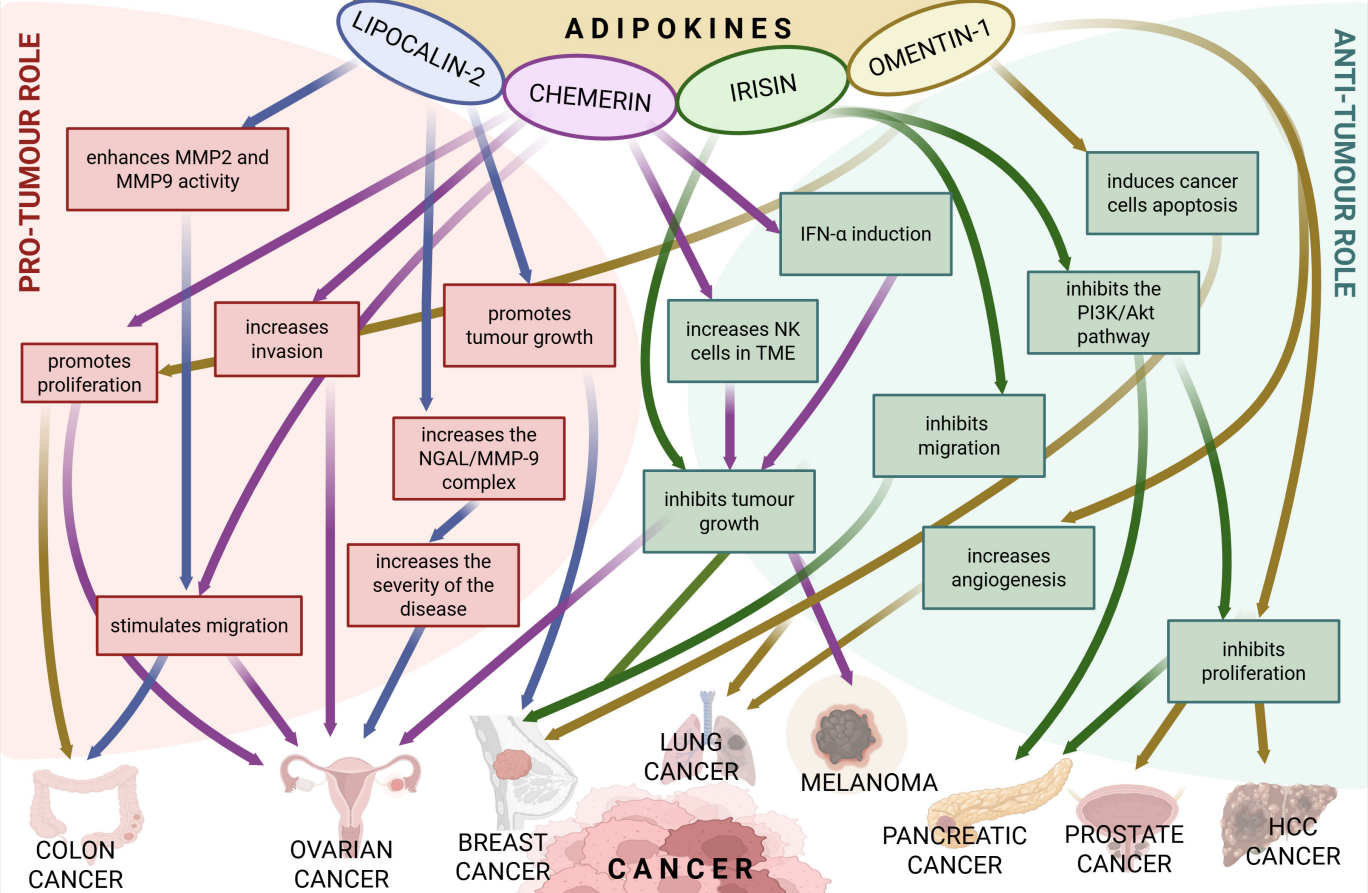
The pro-tumour and anti-tumour role of the analysed adipokines in various types of cancer. Created in https://BioRender.com. AKT – Protein Kinase B, IFNα – Interferon Alpha, MMP2 – Matrix Metalloproteinase-2, MMP9 – Matrix Metalloproteinase-9, PI3K – Phosphoinositide 3-kinase.

Omentin-1 also known as intelectin-1 (ITLN-1) plays an important role in the initiation, progression and promotion of many cancers and influences the intensity of apoptosis in cancer cells (41). Recent studies indicate that significantly elevated omentin concentrations have been found in the development of breast cancer (41, 42), colorectal cancer (43), prostate cancer (44, 45) and pancreatic cancer (46). On the other hand, significantly reduced concentrations of omentin have been found in lung cancer (47) and gynaecological cancers, including ovarian cancer (41). The role of the adipokines under investigation in neoplasm formation is summarised in **Figure 1**.

For this reason, the aim of this study was to conduct the first analysis of the concentration of adipokines – irisin, chemerin, lipocalin-2 and omentin-1 – in the serum and peritoneal fluid of patients with serous ovarian cancer and in the serum of patients with benign ovarian lesions, to determine whether there is a relationship between the concentration of the parameters studied and the degree of histological differentiation of the tumour, and whether there is a relationship between the concentration of the parameters analyzed in serum and peritoneal fluid in patients with serous ovarian cancer, as well as to examine the potential diagnostic usefulness of the parameters analyzed.

## 2 Materials and methods

### 2.1 Study population

The study included 32 female patients with ovarian cancer aged between 36 and 81 years (mean age ± SD; 64.63 ± 12.17 years). These patients were clinically diagnosed and confirmed by histopathological examination with serous ovarian cancer at stage IIIC according to the FIGO (International Federation of Gynaecology and Obstetrics) classification. The diagnosis of ovarian cancer was based on clinical symptoms, gynaecological examination results, laboratory tests and histopathological examination. The FIGO criteria were used to assess the stage of the disease. The histological differentiation of the cancer (grading) was assessed according to the following scale:

- G1 – highly differentiated.
- G2 – moderately differentiated.
- G3 – poorly differentiated.

Considering the above criterion, the percentage of women in the study group is as follows: G1–11 patients (34.4%), G2–10 patients (31.2%), G3–11 patients (34.4%). The material collected for testing was venous blood and peritoneal fluid. The inclusion criteria for the study were: newly diagnosed disease confirmed by histopathological examination, age over 18 years, no pharmacological treatment within the last 3 months prior to sample collection, and informed consent to participate in the study. Exclusion criteria included: coexisting reproductive organ disorders and other chronic diseases, and refusal to participate in the study.

### 2.2 Reference population

The reference group included 16 female patients with diagnosed benign ovarian tumours aged between 19 and 77 years (mean age ± SD; 48.78 ± 12.2 years). The inclusion criteria for the study were: age over 18 years, good general health, no pharmacological treatment in the last 3 months prior to sample collection, and informed consent to participate in the study. Exclusion criteria included: coexisting chronic diseases, especially cancer, and refusal to participate in the study. Venous blood was collected from the patients.

### 2.3 Serum preparation

Blood was collected from patients after clinical diagnosis and before surgery. Blood was collected in the morning from the antecubital vein into a tube containing a clotting activator to obtain serum. Thirty minutes after collection, the tubes containing the material were centrifuged at 1, 500 x g for 15 minutes at room temperature. The serum obtained was separated from the clot, frozen at -80 °C and stored until analysis.

### 2.4 Peritoneal fluid preparation

Peritoneal fluid was collected during laparoscopy for bacteriological examination. The collected material was centrifuged at 1500 x g for 10 minutes at 4 °C. The resulting supernatant was portioned, frozen at -80 °C and stored until analysis.

### 2.5 ELISA tests

The concentration of irisin was indicated using the ELISA (Enzyme-Linked Immunosorbent Assay) test from BioVendor (Brno, Czech Republic). The analytical sensitivity of this test was 1 ng/ml with an intra-assay precision 8.193% and an inter-assay precision 9.719%.

The concentration of chemerin was indicated using the ELISA test from BioVendor (Brno, Czech Republic). The analytical sensitivity of this test was 0.1 ng/ml with an intra-assay precision 7.0% and an inter-assay precision 8.3%.

The concentration of lipocalin-2 was indicated using the ELISA test from BioVendor (Brno, Czech Republic). The analytical sensitivity of this test was 0.02 ng/ml with an intra-assay precision 8.4% and an inter-assay precision 9.8%.

The concentration of omentin-1 was indicated using the ELISA test from BioVendor (Brno, Czech Republic). The analytical sensitivity of this test was 0.5 ng/ml with an intra-assay precision 4.1% and an inter-assay precision 4.8%.

The concentration of CRP (C-reactive protein) was indicated using the ELISA test from apDia, (Raadsherenstraat, Belgium). The analytical sensitivity of this test was 0.225 µg/ml with an intra-assay precision 15.8% and an inter-assay precision 11.7%.

The concentration of CA125 (Carbohydrate Antigen 125) was indicated using the ELISA test from Cloud-Clone Corp., (TX, USA). The analytical sensitivity of this test was 0.58 U/ml with an intra-assay precision <10% and an inter-assay precision <12%.

All assessments were performed in accordance with the manufacturer’s attached protocols.

**Table 1** shows the characteristics of the tests used to perform the current assessments.

**Table 1:** Laboratory characteristics of the tests used for the assays.

Tested parameters	Test name	Producer	Product no.	Test method	Detection range	Sensitivity
Irisin (FNDC5)	Human Irisin ELISA	BioVendor Brno, Czech Republic	RAG018R	Competitive ELISA	0.001-5 µg/ml	1 ng/ml
Chemerin (RARRES2)	Human Chemerin ELISA	BioVendor Brno, Czech Republic	RD191136200R	Sandwich ELISA	0.25–8 ng/ml	0.1 ng/ml
Lipocalin-2 (NGAL)	Human Lipocalin-2/NGAL ELISA	BioVendor Brno, Czech Republic	RD191102200R	Sandwich ELISA	0.3–10 ng/ml	0.02 ng/ml
Omentin-1 (ITLN-1)	Human Omentin-1 ELISA	BioVendor Brno, Czech Republic	RD191100200R	Sandwich ELISA	2–64 g/ml	0.5 ng/ml
C-Reactive Protein (CRP)	CRP ELISA	apDia, Raadsherenstraat, Belgium	740001	Sandwich ELISA	0-100 µg/ml	0.225 µg/ml
CA125	Carbohydrate Antigen 125	Cloud-Clone Corp., TX, USA	SEA154Hu	Sandwich ELISA	1.56–100 U/ml	0.58 U/ml

The following data are from protocols provided by the test manufacturers.

### 2.6 Statistical analysis

The obtained results were subjected to statistical analysis using Statistica 13.3 software (StatSoft Polska Sp. z o.o.). Statistical significance was set at a *p*<0.05.

The normality of the distribution of the studied variables was checked using the Shapiro-Wilk test. In cases where the distribution of the analysed parameters was normal, a parametric ANOVA test was performed. When *p*<0.05, a *post-hoc* test was performed – Tukey’s test for unequal sample sizes. The results obtained were presented using a box plot. However, when the distribution of the analysed parameters was non-normal, a non-parametric Kruskal-Wallis ANOVA was performed. When *p*<0.05, a *post-hoc* test was performed – Dunn’s test. The results obtained were presented using a box plot.

Correlations were tested using Pearson’s rank correlation test (if the data distribution was normal) or Spearman’s test (if the data distribution was non-normal) and presented as a correlation coefficient (r). The level of statistical significance was taken as *p*<0.05. Correlations were classified as negative or positive, and the correlation index (r) was used to define correlations as faint (r<0.1), weak (ranging from 0.1 to 0.3), moderate (ranging from 0.3 to 0.5), strong (ranging from 0.5 to 0.7) or very strong (r>0.7).

Furthermore, a ROC curve was constructed to assess the diagnostic utility of the analysed parameters. Subsequently, the areas under the curve (AUC) were determined and diagnostic performance measures were calculated.

The study was conducted according to the guidelines of the Declaration of Helsinki, and approved by the Ethics Committee of Medical University of Silesia in Katowice, Poland (protocol code KNW/0022/KB1/49/19). All patients agreed to participate in the present study and provided written informed consent.

## 3 Results

The analysis was performed on the concentration of selected adipokines – irisin, chemerin, lipocalin-2 and omentin-1 in serum and peritoneal fluid of patients with ovarian cancer (the study group) and patients with benign ovarian tumours (the reference group). The data obtained from the measurements are presented in **Table 2**.

**Table 2:** Basic descriptive statistics of the analysed parameters.

Parameters	Group	Analysed material	Statistical parameters							
			N	m	SD	Me	Q_1_	Q_3_	x_min_	x_max_
Irisin (ng/ml)	Study group	serum	32	2.591	0.871	2.336	1.924	3.148	1.132	4.815
		peritoneal fluid	32	6.085	1.949	5.431	4.498	7.322	4.067	11.198
	Reference group	serum	16	1.900	0.169	1.893	1.832	1.966	1.574	2.222
Chemerin (ng/ml)	Study group	serum	32	279.369	54.672	280.375	237.550	317.375	190.200	408.600
		peritoneal fluid	32	3.677	1.577	3.622	2.597	4.784	0.358	6.368
	Reference group	serum	16	166.159	45.767	178.050	141.225	198.975	43.350	213.000
Lipocalin-2 (ng/ml)	Study group	serum	32	9.149	4.736	9.442	5.618	13.020	0.943	18.023
		peritoneal fluid	32	11.212	12.041	8.885	3.247	12.325	1.800	51.648
	Reference group	serum	16	1.160	0.384	1.046	0.954	1.097	0.861	2.296
Omentin-1 (ng/ml)	Study group	serum	32	312.119	196.066	270.240	146.445	438.090	48.550	762.780
		peritoneal fluid	32	327.700	261.058	251.925	109.805	531.755	28.450	1101.440
	Reference group	serum	16	413.163	137.226	387.380	320.445	496.530	202.630	664.690
CRP (µg/ml)	Study group	serum	32	47.361	30.521	51.239	16.770	75.706	0.00	88.522
		peritoneal fluid	32	31.877	28.174	30.543	0.00	54.176	0.00	82.001
	Reference group	serum	16	0.738	2.234	0.00	0.00	0.00	0.00	8.905
CA125 (U/ml)	Study group	serum	32	33.836	43.253	20.612	5.476	47.062	0.00	202.291
		peritoneal fluid	32	93.817	59.488	96.769	36.272	147.518	0.591	185.680
	Reference group	serum	16	0.358	1.425	0.00	0.00	0.00	0.00	5.701

N, abundance; m, mean; SD, standard deviation; Me, median; Q1, lower quartile; Q3, upper quartile; x_min_, minimum value; x_max_, maximum value.

Analysed parameters in serum and peritoneal fluid of women with ovarian cancer were assessed according to histological differentiation stage (FIGO).

### 3.1 Irisin

**Figure 2:**
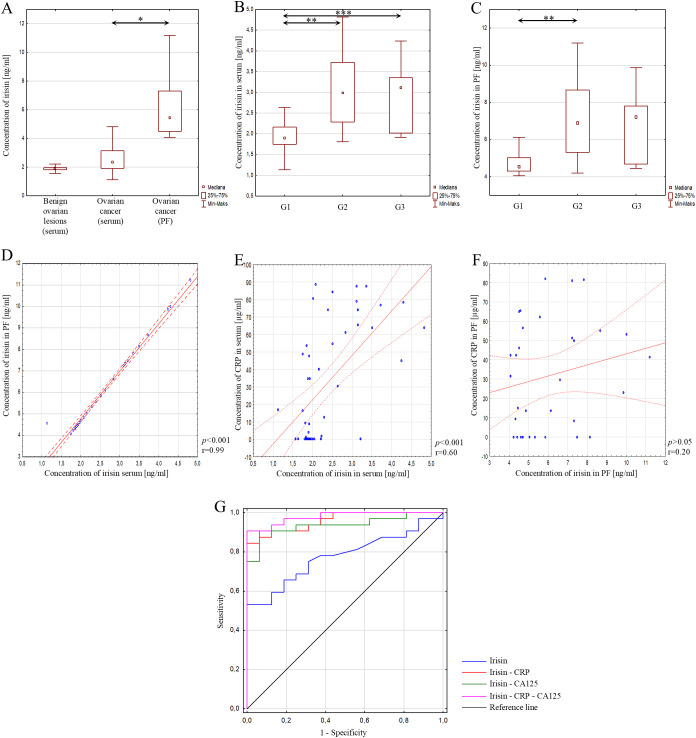
**(A)** Serum irisin concentration in patients with benign ovarian lesions, in serum of patients with ovarian cancer, and in peritoneal fluid of patients with ovarian cancer; **(B)** Serum irisin concentration depending on the degree of histological differentiation of ovarian cancer; **(C)** Irisin concentration in peritoneal fluid depending on the degree of histological differentiation of ovarian cancer; **(D)** Correlation analysis showing the association of irisin between serum and peritoneal fluid; **(E)** Correlation analysis showing the association between irisin and CRP in serum; **(F)** Correlation analysis showing the association between irisin and CRP in peritoneal fluid; **(G)** ROC curve of analysed parameters. In the figure, only data with statistical significance are indicated by arrows. PF - peritoneal fluid, CRP - C-reactive protein, CA125 - Carbohydrate Antigen 125, G1 - highly differentiated, G2 - moderately differentiated, G3 - poorly differentiated, *p<0.001, **p<0.01, ***p<0.05. Solid line - regression line, dotted line - 95% confidence interval, sign ° - values noted during the analysis, *p* - level of statistical significance, r - correlation coefficient.

Statistical analysis did not reveal a statistically significant difference in serum irisin concentration between the study group and the reference group (*p*>0.05), as shown in **Figure 2A**. Next, irisin concentration was assessed according to the degree of histological differentiation of ovarian cancer. This analysis showed a statistically significant difference between grades G1 and G2 (*p*<0.01) and grades G1 and G3 (*p*<0.05), while no significant difference in concentration was found between grades G2 and G3 (*p*>0.05). These results are presented in **Figure 2B**.

In the course of further analysis, irisin concentration in peritoneal fluid was assessed. Statistical analysis showed a statistically significant higher concentration of irisin in peritoneal fluid than in serum in patients with ovarian cancer (*p*<0.001), as shown in **Figure 2A**. In addition, the concentration of the tested parameter was assessed depending on the degree of histological differentiation. The analysis showed a significant difference in irisin concentration only between grades G1 and G2 (*p*<0.01). In other cases, the change in concentration was not statistically significant (*p*>0.05), as shown in **Figure 2C**.

Next, an analysis was conducted to determine the relationship between irisin concentration in serum and peritoneal fluid in patients with ovarian cancer. The analysis showed a statistically significant, positive, very strong correlation between irisin concentration in serum and peritoneal fluid in patients with ovarian cancer (*p*<0.001; r=0.99) (**Figure 2D**). In the next step, a correlation between the concentration of the analysed parameter in serum and peritoneal fluid and the concentration of CRP in serum and peritoneal fluid was investigated. A statistically significant, positive, strong correlation was found between irisin and CRP concentrations in blood serum (*p*<0.001; r=0.60) (**Figure 2E**). However, in peritoneal fluid, the analysis showed no statistically significant correlation between these parameters (**Figure 2F**).

In addition, in order to assess the diagnostic usefulness of the analysed parameter, an analysis was also performed using the ROC curve for irisin and irisin in combination with CRP or CA125, as well as in combination with all these parameters simultaneously. It was found that simultaneous determination of irisin concentration with CRP and CA125 is characterised by better diagnostic efficacy values in patients with ovarian cancer, as evidenced by the obtained AUC results of 0.979, with high sensitivity (91%) and outstanding specificity (100%). The results obtained are presented in **Figure 2G**; **Table 3**.

**Table 3:** Results of the ROC curve analysis of the analysed parameters.

Parameter	AUC	95% CI	Youden index	Cut-Off	Sensitivity	Specificity	PPV	NPV	*P* value
Irisin	0.776	0.648 - 0.905	0.53	2.284	53%	100%	100%	52%	<0.001
CRP	0.924	0.846 - 1.000	0.84	9.320	84%	100%	100%	76%	<0.001
CA125	0.906	0.821 - 0.991	0.78	2.298	84%	94%	96%	75%	<0.001
Irisin - CRP	0.959	0.911 - 1.000	0.84	11.130	84%	100%	100%	76%	<0.001
Irisin - CA125	0.938	0.869 - 1.000	0.84	2.630	91%	94%	97%	83%	<0.001
Irisin - CRP - CA125	0.979	0.946 - 1.000	0.91	11.131	91%	100%	100%	84%	<0.001

AUC, area under the ROC curve; PPV, positive predictive values; NPV, negative predictive values.

### 3.2 Chemerin

**Figure 3:**
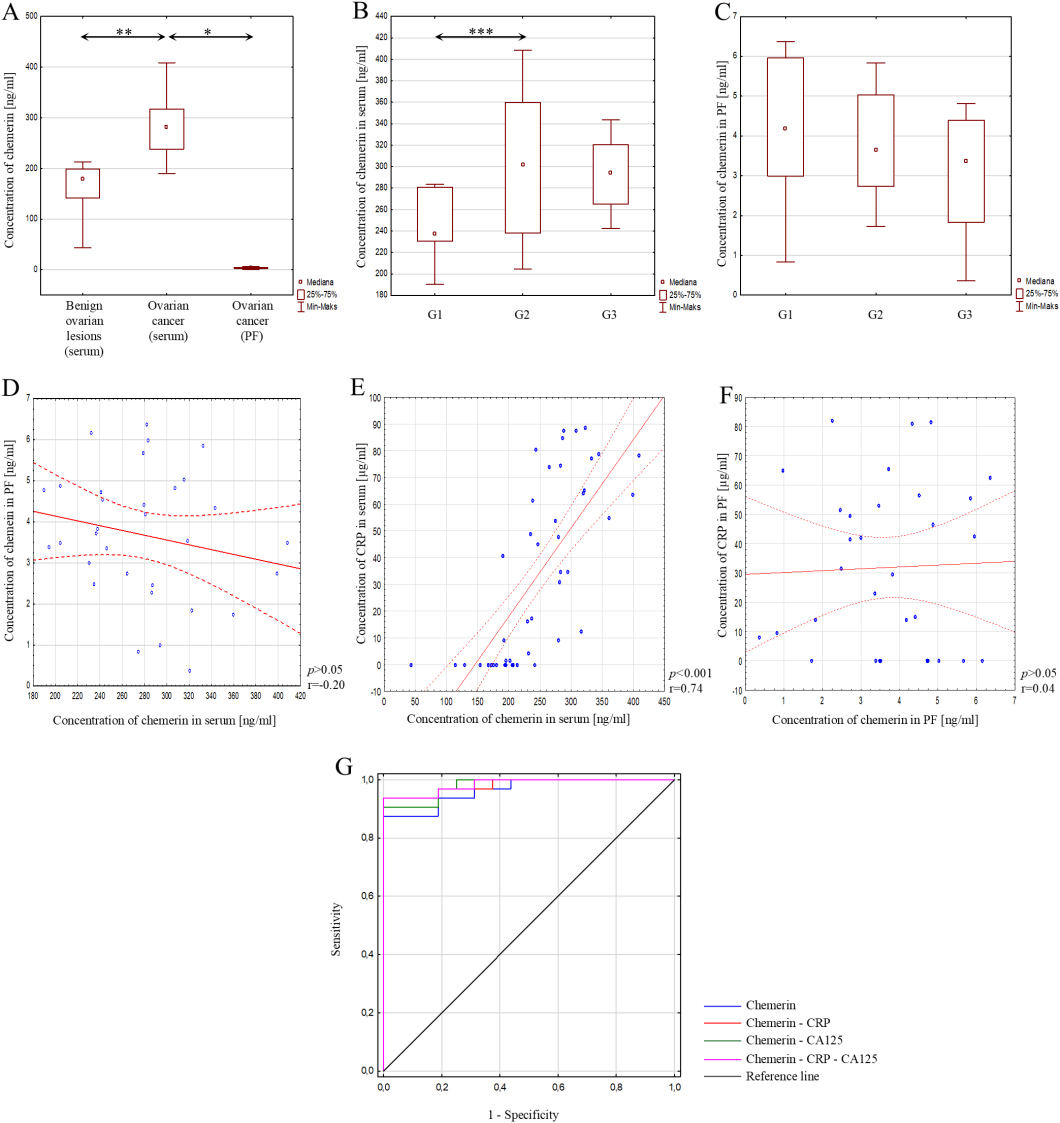
**(A)** Serum chemerin concentration in patients with benign ovarian lesions, in serum of patients with ovarian cancer, and in peritoneal fluid of patients with ovarian cancer; **(B)** Serum chemerin concentration depending on the degree of histological differentiation of ovarian cancer; **(C)** Chemerin concentration in peritoneal fluid depending on the degree of histological differentiation of ovarian cancer; **(D)** Correlation analysis showing the association of chemerin between serum and peritoneal fluid; **(E)** Correlation analysis showing the association between chemerin and CRP in serum; **(F)** Correlation analysis showing the association between chemerin and CRP in peritoneal fluid; **(G)** ROC curve of analysed parameters. In the figure, only data with statistical significance are indicated by arrows. PF - peritoneal fluid, CRP - C-reactive protein, CA125 - Carbohydrate Antigen 125, G1 - highly differentiated, G2 - moderately differentiated, G3 - poorly differentiated, *p<0.001, **p<0.01, ***p<0.05. Solid line - regression line, dotted line - 95% confidence interval, sign ° - values noted during the analysis, *p* - level of statistical significance, r - correlation coefficient.

The statistical analysis showed significantly higher serum chemerin concentrations in patients with ovarian cancer compared to serum chemerin concentrations in patients with benign ovarian lesions (*p*<0.01) (**Figure 3A**). The concentration of chemerin was also analysed depending on the degree of histological differentiation of ovarian cancer. Only a statistically significant difference in chemerin concentration was found between grades G1 and G2 (*p*<0.05), as shown in **Figure 3B**.

In the process of further analysis, the concentration of chemerin in peritoneal fluid was assessed in patients with ovarian cancer. The analysis showed a statistically significant lower concentration of chemerin in peritoneal fluid compared to the concentration of chemerin in the serum of patients with ovarian cancer (*p*<0.001) (**Figure 3A**). When analysing chemerin concentration in peritoneal fluid depending on the histological differentiation of ovarian cancer, no statistically significant difference was observed between grades G1, G2 and G3 (*p*>0.05), as shown in **Figure 3C**.

In the next step, a correlation test was performed to check whether there was a correlation between chemerin concentration in serum and peritoneal fluid in patients with ovarian cancer. The analysis showed no correlation between chemerin concentration in serum and peritoneal fluid in patients with ovarian cancer (*p*>0.05; r=-0.20) (**Figure 3D**). Next, the concentration of the analysed parameter in serum and peritoneal fluid was correlated with the concentration of CRP in serum and peritoneal fluid. A statistically significant, positive, very strong correlation was found between the concentration of chemerin and CRP in blood serum (*p*<0.001; r=0.74) (**Figure 3E**). In contrast, in peritoneal fluid, the analysis showed no statistically significant correlation between the assessed parameters (*p*>0.05; r=0.04) (**Figure 3F**).

The potential diagnostic usefulness of chemerin measurement in patients with ovarian cancer was also investigated. For this purpose, an analysis was performed using a ROC curve for chemerin and for chemerin in combination with CRP or CA125, as well as with these parameters simultaneously. It was shown that simultaneous measurement of chemerin, CRP and CA125 concentrations is characterised by good diagnostic efficacy values, with AUC = 0.984, 94% sensitivity and 100% specificity. These results are presented in **Figure 3G**; **Table 4**.

**Table 4:** Results of the ROC curve analysis of the analysed parameters.

Parameter	AUC	95% CI	Youden index	Cut-Off	Sensitivity	Specificity	PPV	NPV	*P* value
Chemerin	0.965	0.921 - 1.000	0.88	230.500	86%	100%	100%	80%	<0.001
CRP	0.924	0.846 - 1.000	0.84	9.320	84%	100%	100%	76%	<0.001
CA125	0.906	0.821 - 0.991	0.78	2.298	84%	94%	96%	75%	<0.001
Chemerin - CRP	0.977	0.943 - 1.000	0.91	230.620	91%	100%	100%	84%	<0.001
Chemerin - CA125	0.980	0.951 - 1.000	0.91	230.300	91%	100%	100%	84%	<0.001
Chemerin - CRP - CA125	0.984	0.958 - 1.000	0.94	230.300	94%	100%	100%	89%	<0.001

AUC, area under the ROC curve; PPV, positive predictive values; NPV, negative predictive values.

### 3.3 Lipocalin-2

**Figure 4:**
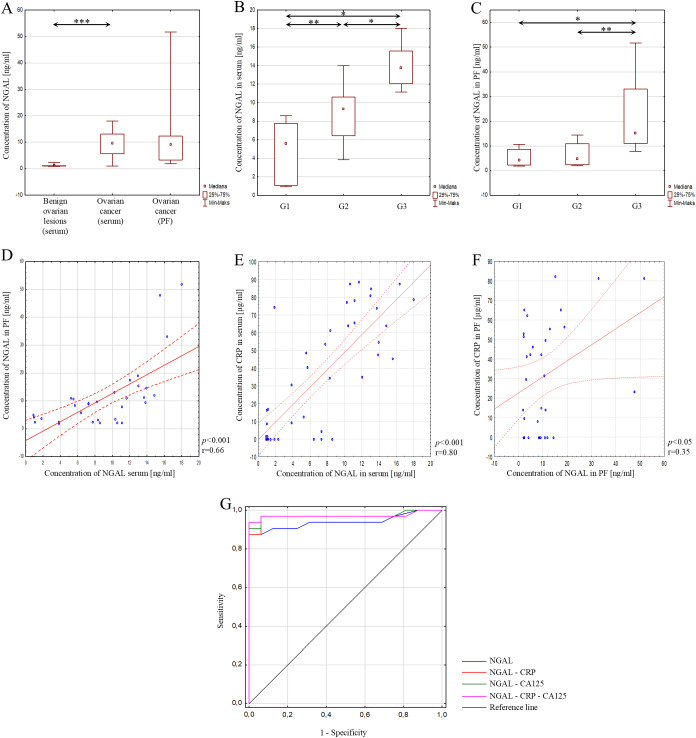
**(A)** Serum NGAL concentration in patients with benign ovarian lesions, in serum of patients with ovarian cancer, and in peritoneal fluid of patients with ovarian cancer; **(B)** Serum NGAL concentration depending on the degree of histological differentiation of ovarian cancer; **(C)** NGAL concentration in peritoneal fluid depending on the degree of histological differentiation of ovarian cancer; **(D)** Correlation analysis showing the association of NGAL between serum and peritoneal fluid; **(E)** Correlation analysis showing the association between NGAL and CRP in serum; **(F)** Correlation analysis showing the association between NGAL and CRP in peritoneal fluid; **(G)** ROC curve of analysed parameters. In the figure, only data with statistical significance are indicated by arrows. PF - peritoneal fluid, NGAL - lipocalin-2, CRP - C-reactive protein, CA125 - Carbohydrate Antigen 125, G1 - highly differentiated, G2 - moderately differentiated, G3 - poorly differentiated, *p < 0.001, **p < 0.01, ***p < 0.05. Solid line - regression line, dotted line - 95% confidence interval, sign ° - values noted during the analysis, *p* - level of statistical significance, r - correlation coefficient.

The next parameter analysed was lipocalin-2. The analysis showed that the concentration of lipocalin-2 was statistically significantly higher in the serum of patients with ovarian cancer than in patients with benign ovarian lesions, where *p*<0.001, as shown in **Figure 4A**. The concentration of NGAL in the group of patients with ovarian cancer was also analysed depending on the degree of histological differentiation of the tumour. A statistically significant difference in NGAL concentration was found between grades: G1 and G2 (*p*<0.01), G1 and G3 (*p*<0.001) and G2 and G3 (*p*<0.001). These results are presented in **Figure 4B**.

NGAL concentrations in peritoneal fluid were also assessed in patients with ovarian cancer. Statistical analysis showed no statistically significant difference in NGAL concentrations in serum and peritoneal fluid in patients with ovarian cancer (*p*>0.05), as shown in **Figure 4A**. In addition, NGAL concentrations in peritoneal fluid were also assessed according to the histological differentiation grade of ovarian cancer. Statistically significant higher concentrations were found in grade G3 compared to grade G1 (*p*<0.001) and grade G2 (*p*<0.01). The analysis did not reveal any significant difference between G1 and G2 (*p*>0.05), as shown in **Figure 4C**.

The study also examined whether there is a correlation between NGAL concentrations in serum and peritoneal fluid in patients with ovarian cancer. The analysis showed a statistically significant, positive, strong correlation between NGAL concentrations in serum and peritoneal fluid in patients with ovarian cancer (*p*<0.001; r=0.66) (**Figure 4D**). Next, serum and peritoneal fluid NGAL concentrations were correlated with CRP concentrations in serum and peritoneal fluid. The correlation analysis showed a statistically significant, positive, very strong correlation between serum NGAL concentrations in patients with ovarian cancer (*p*<0.001; r=0.80) (**Figure 4E**), while in the case of peritoneal fluid, the correlation analysis showed a statistically significant, positive, moderate correlation (*p*<0.05; r=0.35) (**Figure 4F**).

The diagnostic usefulness of NGAL measurement in patients with ovarian cancer was also evaluated. ROC curve analysis showed that the best indicators of diagnostic effectiveness are simultaneous NGAL, CRP and CA125 measurements, as opposed to single measurements of these parameters, as evidenced by the results obtained: AUC=0.972, 94% sensitivity and 100% specificity. The results obtained are presented in **Figure 4G**; **Table 5**.

**Table 5:** Results of the ROC curve analysis of the analysed parameters.

Parameter	AUC	95% CI	Youden index	Cut-Off	Sensitivity	Specificity	PPV	NPV	*P* value
NGAL	0.940	0.871 - 1.000	0.88	3.855	86%	100%	100%	80%	<0.001
CRP	0.924	0.846 - 1.000	0.84	9.320	84%	100%	100%	76%	<0.001
CA125	0.906	0.821 - 0.991	0.78	2.298	84%	94%	96%	75%	<0.001
NGAL - CRP	0.970	0.920 - 1.000	0.91	6.440	97%	94%	97%	94%	<0.001
NGAL - CA125	0.972	0.923 - 1.000	0.91	7.300	91%	100%	100%	84%	<0.001
NGAL - CRP - CA125	0.972	0.920 - 1.000	0.94	12.983	94%	100%	100%	89%	<0.001

AUC, area under the ROC curve; PPV, positive predictive values; NPV, negative predictive values.

### 3.4 Omentin-1

**Figure 5:**
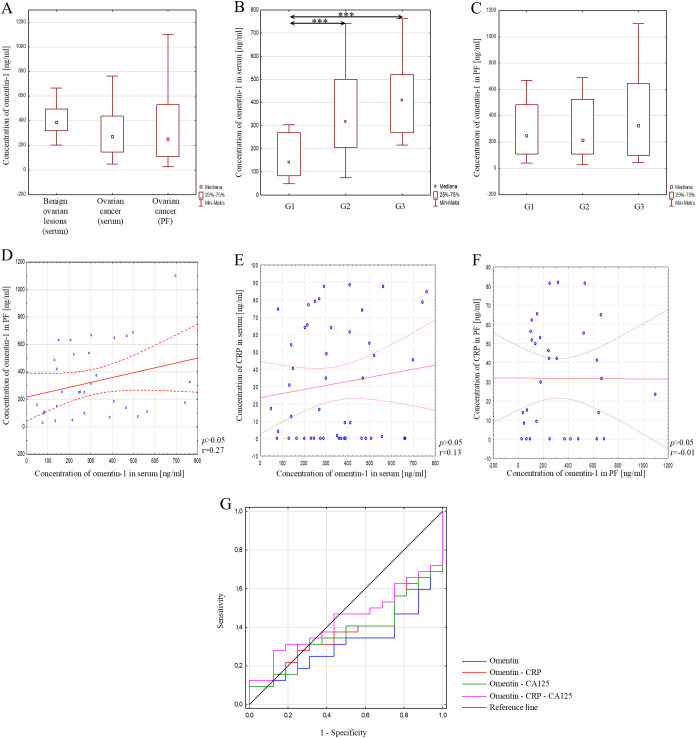
**(A)** Serum omentin-1 concentration in patients with benign ovarian lesions, in serum of patients with ovarian cancer, and in peritoneal fluid of patients with ovarian cancer; **(B)** Serum omentin-1 concentration depending on the degree of histological differentiation of ovarian cancer; **(C)** Omentin-1 concentration in peritoneal fluid depending on the degree of histological differentiation of ovarian cancer; **(D)** Correlation analysis showing the association of omentin-1 between serum and peritoneal fluid; **(E)** Correlation analysis showing the association between omentin-1 and CRP in serum; **(F)** Correlation analysis showing the association between omentin-1 and CRP in peritoneal fluid; **(G)** ROC curve of analysed parameters. In the figure, only data with statistical significance are indicated by arrows. PF - peritoneal fluid, CRP - C-reactive protein, CA125 - Carbohydrate Antigen 125, G1 - highly differentiated, G2 - moderately differentiated, G3 - poorly differentiated, *p<0.001, **p<0.01, ***p<0.05. Solid line - regression line, dotted line - 95% confidence interval, sign ° - values noted during the analysis, *p* - level of statistical significance, r - correlation coefficient.

The concentration of omentin-1 was also assessed. The analysis showed no statistically significant difference in serum omentin-1 concentration between the reference group and the study group (*p*>0.05) (**Figure 5A**). In addition, serum concentrations of the analysed parameter were analysed according to the degree of histological differentiation of ovarian cancer. The analysis showed a statistically significant higher concentration in grades G2 and G3 compared to grade G1, where the p-value in both cases was *p*<0.05. However, no statistically significant difference was found between grades G2 and G3 (*p*>0.05). These data are illustrated in **Figure 5B**.

Next, the concentration of omentin-1 in peritoneal fluid was assessed. Statistical analysis showed no statistically significant difference in the concentration of omentin-1 in peritoneal fluid compared to the concentration of this parameter in serum in patients with ovarian cancer (*p*>0.05) (**Figure 5A**). Further analysis also showed no statistically significant difference in the concentration of the analysed parameter in peritoneal fluid depending on the degree of histological differentiation of ovarian cancer, as shown in **Figure 5C**.

In the next step, a correlation test was performed to check whether there was a relationship between the concentration of omentin-1 in serum and peritoneal fluid in patients with ovarian cancer. The analysis showed no correlation between the concentration of omentin-1 in the analysed materials (*p*>0.05; r=0.27) (**Figure 5D**). Furthermore, statistical analysis showed no correlation between serum omentin-1 and CRP concentrations (*p*>0.05; r=0.13) (**Figure 5E**) and the concentration of these parameters in peritoneal fluid (*p*>0.05; r=-0.01) (**Figure 5F**).

The diagnostic usefulness of the analysed parameter was also verified. Analysis using the ROC curve showed that simultaneous determination of omentin-1, CRP and CA125 has better diagnostic parameters than the determination and analysis of these parameters individually, but the results obtained are not satisfactory. A relatively low area under the curve (AUC=0.438) and low sensitivity of 28% were obtained, with a fairly good specificity of 88%. The results obtained are presented in **Figure 5G**; **Table 6**.

**Table 6:** Results of the ROC curve analysis of the analysed parameters.

Parameter	AUC	95% CI	Youden index	Cut-Off	Sensitivity	Specificity	PPV	NPV	*P* value
Omentin	0.316	0.168 - 0.465	0.09	697.720	10%	100%	100%	36%	<0.05
CRP	0.924	0.846 - 1.000	0.84	9.320	84%	100%	100%	76%	<0.001
CA125	0.906	0.821 - 0.991	0.78	2.298	84%	94%	96%	75%	<0.001
Omentin - CRP	0.375	0.219 - 0.531	0.09	742.780	9%	100%	100%	36%	>0.05
Omentin - CA125	0.371	0.215 - 0.527	0.09	706.562	9%	100%	100%	36%	>0.05
Omentin - CRP - CA125	0.438	0.278 - 0.597	0.16	560.865	28%	88%	82%	38%	>0.05

AUC, area under the ROC curve; PPV. positive predictive values; NPV, negative predictive values.

### 3.5 CA125 and CRP

In addition, serum CA125 and CRP concentrations in serum and peritoneal fluid were analysed.

**Figure 6:**
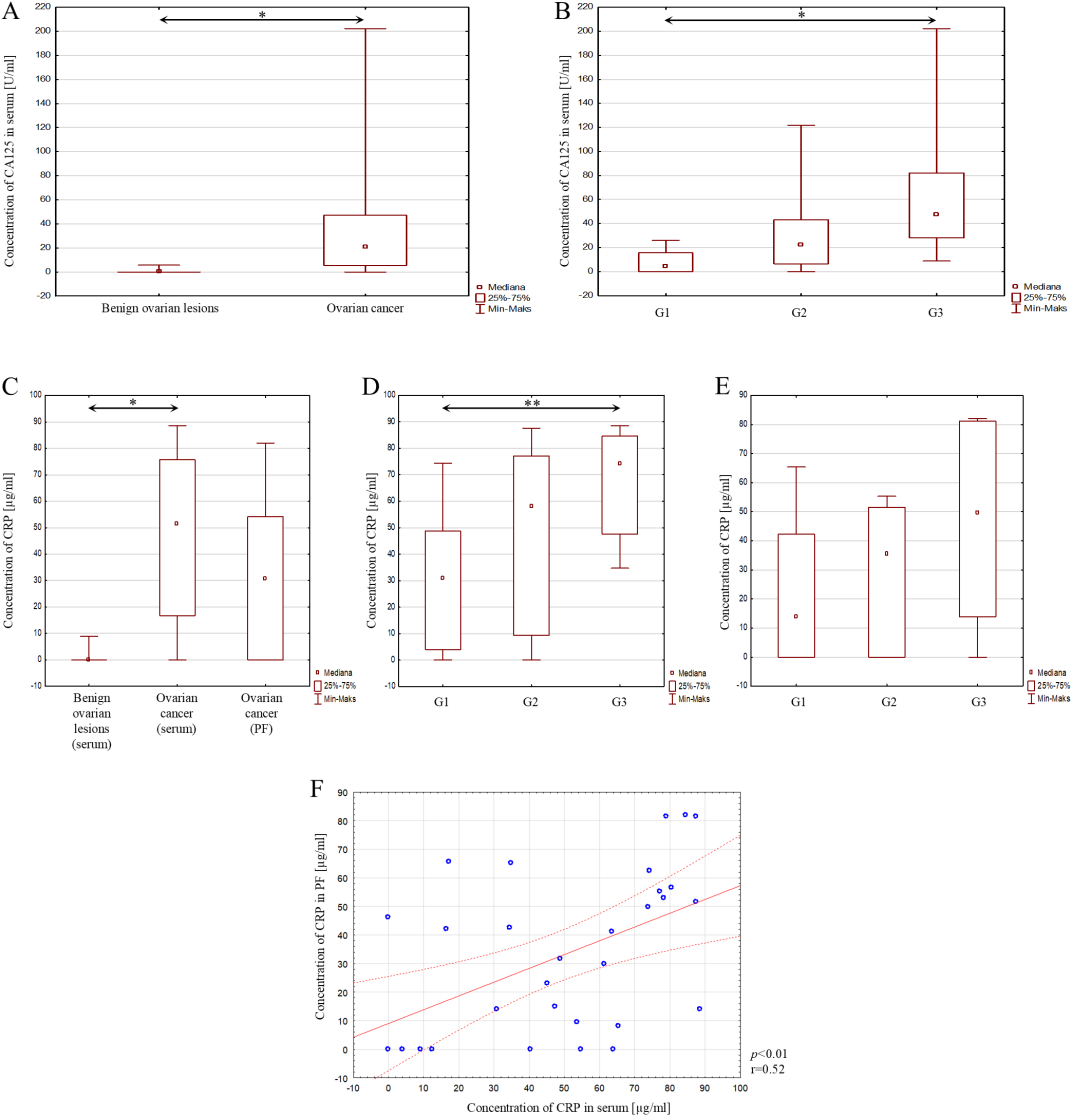
**(A)** Serum CA125 concentration in patients with benign ovarian lesions and with ovarian cancer; **(B)** Serum CA125 concentration depending on the degree of histological differentiation of ovarian cancer; **(C)** Serum CRP concentration in patients with benign ovarian lesions, in serum of patients with ovarian cancer, and in peritoneal fluid of patients with ovarian cancer; **(D)** Serum CRP concentration depending on the degree of histological differentiation of ovarian cancer; **(E)** CRP concentration in peritoneal fluid depending on the degree of histological differentiation of ovarian cancer; **(F)** ROC curve of analysed parameters. In the figure, only data with statistical significance are indicated by arrows. PF - peritoneal fluid, CRP - C-reactive protein, CA125 - Carbohydrate Antigen 125, G1 - highly differentiated, G2 - moderately differentiated, G3 - poorly differentiated, *p<0.001, **p<0.01, ***p<0.05. Solid line - regression line, dotted line - 95% confidence interval, sign ° - values noted during the analysis, *p* - level of statistical significance, r - correlation coefficient.

Significantly elevated serum CA125 concentrations were found in patients with ovarian cancer compared to patients with benign ovarian lesions, with a difference of *p*<0.001, as shown in **Figure 6A**. A statistically significant difference in the concentration of this marker was also observed only between the histological differentiation grades G1 and G3 of ovarian cancer (*p*<0.001), as shown in **Figure 6B**.

Next, serum CRP concentrations were assessed. Statistical analysis showed a statistically significant higher CRP concentration in the group of patients with ovarian cancer compared to the group of patients with benign ovarian lesions, and this difference was equal to *p*<0.001 (**Figure 6C**). Taking into account the degrees of histological differentiation of ovarian cancer, statistical analysis showed only a significant difference between grades G1 and G3 (*p*<0.01), as shown in **Figure 6D**.

In the next step, CRP concentration in peritoneal fluid was assessed. In the group of patients with ovarian cancer, statistical analysis did not show a significant difference in CRP concentration in serum and peritoneal fluid in patients from the study group, as shown in **Figure 6E**.

Furthermore, no significant difference in CRP concentration was found depending on the histological differentiation of ovarian cancer. However, the correlation test showed a statistically significant, positive, strong correlation between CRP concentration in serum and peritoneal fluid in patients with ovarian cancer (*p*<0.01; r=0.52). This result is presented in **Figure 6F**.

## 4 Discussion

Ovarian cancer continues to be characterised by high morbidity and mortality rates. Due to the non-specific symptoms accompanying the development of OC, most cases of ovarian cancer are diagnosed at an advanced clinical stage. Despite numerous studies, it has not yet been possible to develop a biomarker with a high degree of sensitivity and specificity. Therefore, the identification of new ovarian cancer biomarkers is crucial for the early detection and diagnosis of ovarian cancer, which will allow for faster implementation of targeted treatment and increase patient survival rates (48–50).

The results of previous reports indicate that chronic inflammation is a factor in the development of many cancers, including ovarian cancer. This environment promotes the secretion of various pro-inflammatory mediators, of which adipokines are a particularly important group (8, 9, 15).

There are few studies available on the role of irisin in ovarian cancer. Zhu et al. (51, 52) demonstrated that irisin inhibits the proliferation, invasion and migration of epithelial ovarian cancer by regulating EMT in the PI3K/Akt signalling pathway. Furthermore, the authors found that irisin expression in epithelial ovarian cancer tissue was significantly higher than in physiological ovarian tissue. Furthermore, the level of irisin expression in patients with late clinical stage disease with lymph node metastases was lower than in patients with early clinical stage disease without metastases. According to the authors, irisin/FNDC5 can be used as a marker to predict potential tumour metastasis, assess prognosis and develop new potential therapeutic agents for the treatment of OC. Similar studies were conducted by Kuloglu et al. (53), who performed immunohistochemical analysis of human ovarian, breast and cervical cancer tissues. The authors demonstrated increased immunoreactivity of irisin in tissues obtained from breast, ovarian, cervical and endometrial hyperplasia compared to controls and suggest a key role for irisin in the carcinogenesis of the tumours studied. Interesting observations are also provided by studies conducted by Alizadeh Zarei et al. (54) also provide interesting observations, demonstrating that irisin inhibited the proliferation of ovarian cancer cells in a time- and dose-dependent manner, effectively reduced the tendency of cancer cells to migrate, caused apoptosis of cancer cells, and inhibited the malignant nature of ovarian cancer cells via the HIF-1α (Hypoxia-Inducible Factor 1-Alpha) pathway. No studies have been conducted to date on the analysis of irisin concentration in patients with ovarian cancer. Such studies were conducted for the first time as part of this work, both in serum and peritoneal fluid in patients with ovarian cancer. The analysis did not reveal any significant changes in irisin concentration between the study and control groups, but the results of the analysis of adipokine concentration between histological differentiation grades in ovarian cancer patients provided interesting observations. Statistically significant changes in concentration between grades G1 and G2 and grades G1 and G3 in both serum and peritoneal fluid indicate the involvement of irisin in tumour development. Furthermore, the statistically significant higher concentration of irisin in the peritoneal fluid of ovarian cancer patients compared to the concentration in serum indicates increased secretion of this adipokine in the tumour microenvironment. In contrast, the analysis of the diagnostic effectiveness of irisin in the diagnosis of ovarian cancer in our study showed excellent specificity (100%) but relatively low sensitivity (53%), making it a weaker biomarker compared to CRP or CA125. This analysis shows that the combination of individual biomarkers (irisin, CRP and CA125) has excellent sensitivity and specificity rates of 91% and 100%, respectively. Available studies on the role of chemerin in the pathogenesis of ovarian cancer are inconclusive (15). Most of the results obtained show the pro-tumour nature of chemerin in ovarian cancer (55–58). Other observations indicate the opposite effect. Studies by Gao et al. (55) showed that chemerin promotes the proliferation and migration of ovarian cancer through increased PD-L1 expression. Similar results were obtained by Sun and Guo (58), who showed that chemerin is involved in the proliferation, migration and invasion of ovarian cancer cells. According to the authors, these processes occur with the participation of the CMKLR1/RhoA/ROCK1 (Chemerin Chemokine-like Receptor 1/Ras Homolog Gene Family Member A/Rho-Associated Kinase) dependent EMT pathway. For the first time, simultaneous analysis of chemerin concentration in serum and peritoneal fluid was performed in patients with ovarian cancer. The analysis showed significantly higher serum chemerin concentrations in ovarian cancer patients compared to the control group and between histological differentiation grades G1 and G2, indicating the involvement of this adipokine in cancer development. Furthermore, the statistically significant lower concentration of chemerin in the peritoneal fluid of patients with ovarian cancer compared to the concentration in serum indicates an intensification of the systemic effect, and chemerin in serum may also be secreted from another source.

Zhao et al. (57) obtained different results, showing that the concentration of proteolytic forms of chemerin in peritoneal fluid was significantly higher than in the other groups studied, which, according to the authors, indicates the inflammatory nature of ascites. Furthermore, according to the authors, chemerin enhances the growth of ovarian tumours.

Other *in silico* studies conducted by Weber et al. (56) in physiological ovarian tissue, ovarian cancer and ovarian cancer metastases indicate a decrease in mRNA expression of the chemerin gene (RARRES2) in both cancer and metastatic tissue compared to physiological ovarian tissue. In *in vitro* studies, Schmitt et al. (59) demonstrated the anticancer activity of chemerin. The authors indicate that the main mechanism of this activity is the induction of IFNα (Interferon Alpha) anticancer response genes, resulting from an increase in the secretion of this cytokine induced by chemerin.

Interestingly, the assessment of the diagnostic usefulness of chemerin testing in ovarian cancer patients showed very good sensitivity and specificity rates, better than the commonly used CA125 marker. However, the combination of chemerin-CRP-CA125 resulted in excellent test evaluation values, with sensitivity equal to 94% and specificity equal to 100%, indicating that the combination of these biomarkers is a better diagnostic tool than the determination of these parameters separately.

The significance of lipocalin-2 in tumour progression and chemoresistance is multifaceted, as it can act pro- or anti-tumour depending on the type of cancer, and its expression correlates with the stage and aggressiveness of the disease (60). Cho and Kim (40) used real-time PCR (Polymerase Chain Reaction) and immunohistochemical analysis to demonstrate increased NGAL expression in patients with ovarian cancer, borderline tumours and benign tumours compared to normal tissue. In contrast, Gupta et al. (37) analysed the NGAL/MMP-9 complex, which is involved in the development and progression of many cancers. The authors pointed to a significantly higher concentration of this complex in the serum of patients with ovarian cancer. In addition, a higher concentration of this complex correlated with the stage of epithelial ovarian cancer. In contrast, Zhao et al. (39) showed that lncRNA (Long Noncoding RNA) TMPO-AS1 (TMPO antisense RNA 1) is capable of activating LCN2 transcription with the participation of E2F6, thereby influencing the migration, invasion and angiogenesis of ovarian cancer cells. Importantly, these studies indicate a potential therapeutic target through the inhibition of lncRNA TMPO-AS1. To date, only a few studies have been conducted showing that the expression of immunoreactive NGAL (irNGAL) in ovarian tumours changes with disease staging and that this change is reflected in peripheral blood NGAL concentrations. irNGAL was not present in normal ovaries, and NGAL expression was weak to moderate in benign tissues. Compared to control samples, NGAL concentrations were 2- and 2.6-fold higher in patients with benign tumours and grade 1 tumours (*p*>0.05). The authors suggested the potential role of NGAL as a marker for early-stage ovarian cancer (38).

The analysis of lipocalin-2 concentration showed significantly higher levels of this parameter in the serum of patients with ovarian cancer compared to the serum of women in the control group. The analysis of adipokine concentrations between histological differentiation grades in serum and peritoneal fluid in patients with ovarian cancer also provided interesting observations. Statistically significant changes in concentration between grades G1 and G2, G1 and G2, and G1 and G3 indicate the involvement of NGAL in tumor development. No statistical significance was found between NGAL concentrations in serum and peritoneal fluid, but a statistically significant positive correlation was found between NGAL concentrations in serum and peritoneal fluid in patients with ovarian cancer, which may suggest the existence of a disturbed regulatory mechanism involving this adipokine, modulated by interactions between immune system cells and cancer cells.

As in the case of irisin and chemerin, NGAL also showed very good diagnostic performance parameters, with sensitivity and specificity of 86% and 100%, respectively. In addition, combining NGAL assessment with CRP and CA125 resulted in an increase in sensitivity to 94% and maintained specificity at 100%. Therefore, the simultaneous analysis of these parameters makes them good indicators in the diagnosis of ovarian cancer.

To date, few studies have been conducted to investigate the role of omentin in ovarian cancer. Au-Yeung et al. (20) determined circulating ITLN-1 levels in serum samples obtained from healthy women, as well as preoperative serum samples from patients with HGSC (high-grade serous carcinoma) and from individuals with benign gynecological diseases. They showed that circulating ITLN-1 levels were significantly lower in patients with HGSC than in healthy women or individuals with benign gynecological diseases, suggesting that the presence of OC cells may reduce circulating ITLN-1 levels. According to the authors, therapeutic strategies developed to increase ITLN-1 regulation in OC patients may inhibit OC progression and metastasis and improve patient survival rates.

The study did not reveal any statistically significant differences between the serum concentrations of omentin-1 in patients with ovarian cancer and those in the control group. Although the differences were not statistically significant, the observed changes in concentrations may suggest the involvement of adipokines in interactions between immune system cells in cancer and in benign tumors. In addition, the highest concentration of omentin-1 was found in the serum of patients with G3 differentiation, which was statistically significant compared to G1, which may indicate the involvement of this adipokine in the pathogenesis of ovarian cancer.

Although our results contradict previous reports, differences in the metabolic phenotype of the women studied should be taken into account. In our cohort, the average age was higher, which often correlates with changes in fat redistribution and a different metabolic status than in younger patients, which in turn directly affects circulating adipokine concentrations. No statistically significant changes in the concentration of the studied parameter were observed in the peritoneal fluid of patients with ovarian cancer, which may suggest the existence of compensatory regulatory mechanisms occurring in the tumor microenvironment. Due to the limited amount of data, future studies should aim to better understand the role of ITLN-1 in this cancer.

By analyzing the concentration of omentin-1 in terms of diagnostic usefulness, this study shows that omentin-1 measurement has ideal specificity (100%), but very poor sensitivity of only 10%. When combining the assessment and analysis of omentin-1, CRP, and CA125, sensitivity increases slightly to 28%, but unfortunately specificity decreases to 88%. Therefore, the determination of omentin-1 as a single biomarker or in combination with CRP and CA125 does not make it a good marker useful in the diagnosis of ovarian cancer.

## 5 Study limitations

Despite the promising results obtained for the adipokine profile (irisin, chemerin, lipocalin-2 and omentin-1) in ovarian cancer, this study has several important limitations that should be taken into account when interpreting the data.

The study was conducted on a relatively small group of patients (study group n=32 vs. reference group n=16), which limits the statistical power of the analysis and increases the risk of error resulting from the presence of outliers. Furthermore, the study cohort was limited exclusively to patients with advanced serous carcinoma (FIGO IIIC). Such high homogeneity of the group allows for a precise assessment of the advanced stage, but prevents the generalisation of the results to early stages of the cancer and other histological subtypes. On the other hand, although the lack of representation of early clinical stages limits the potential application of the studied markers in screening tests, our analysis provides important data on disease progression. The precise characterisation of stage IIIC allows the identification of metabolic parameters correlating with high tumour aggressiveness and inflammation.

The main limitation is the lack of detailed data on body mass index (BMI) and the full metabolic status of the patients included in the study. Since adipokines such as omentin-1 and chemerin are closely related to the amount and activity of adipose tissue, it cannot be completely excluded that the observed differences are partly modulated by the baseline metabolic status of the patients and not solely by the neoplastic process.

Due to the bimodal distribution of data, some parameters, such as ROC analysis or correlation coefficients, may show a tendency to overfitting. The very high AUC values and strong correlations (r=0.99) obtained should be interpreted as pilot results requiring validation in a larger, independent cohort.

The altered adipokine profile may be part of a broader systemic response to the ongoing cancer process and not necessarily a factor initiating carcinogenesis. Furthermore, the demonstrated correlations between adipokine concentrations and CRP concentrations suggest a link with the systemic inflammatory response. However, in the absence of direct measurements of immunological parameters, conclusions about the specific immunomodulatory mechanisms of the adipokines studied remain hypothetical and require further additional research.

## 6 Conclusion

In this study, for the first time, the concentration of new adipokines: irisin, chemerin, lipocalin-2, and omentin-1 in patients with serous ovarian cancer were simultaneously assessed in both serum and peritoneal fluid for the first time, but further intensive research on a larger study group seems necessary to better understand the role of these adipokines in the development of ovarian cancer and to determine their potential role in diagnosis, prognosis, and treatment.

1. Adipokines studied: irisin, chemerin, lipocalin-2, and omentin-1 are involved in interactions between serous ovarian cancer cells and immune system cells, which on the one hand may enhance the antitumor response, but on the other hand may also promote cancer progression, making them particularly interesting proteins in the context of potential clinical application.
2. Changes in the concentration of irisin and chemerin in the peritoneal fluid microenvironment may indicate the involvement of these adipokines in the local immune response.
3. The combination of irisin, chemerin, and lipocalin-2 with CRP and CA125 significantly increases diagnostic efficiency compared to the single determination of these parameters, suggesting that panel assessment is a potential tool to aid in the diagnosis of this disease entity.

## Data Availability

The raw data supporting the conclusions of this article will be made available by the authors, without undue reservation.
